# Preoperative prediction of acute severe cholecystitis using an attentive interpretable tabular network (TabNet)-based radiomics model and a stacking ensemble: a two-center study

**DOI:** 10.1080/07853890.2026.2732512

**Published:** 2026-09-15

**Authors:** Hong-Yu Long, Wei Li, Xin Yan, Bai-Qing Chen, Feng Xie

**Affiliations:** ^a^ Department of Interventional Medicine, Liaoning Provincial Institute of Geriatrics, Shenyang, China; ^b^ Department of Interventional Medicine, Jinqiu Hospital of Liaoning province, Shenyang, China; ^c^ Department of Radiology, Panjin Liaohe Oilfield Gem Flower Hospital, Panjin, China; ^d^ Department of Imaging 1, The Rehabilitation Hospital of Shaanxi Province, Xi’an, China; ^e^ Department of Radiology, The People’s Hospital of Liaoning Province, Shenyang, China

**Keywords:** Acute cholecystitis, suppuration, gangrenous, computed tomography, deep learning

## Abstract

**Objectives:**

To develop an explainable and cost-effective predictive model for acute severe cholecystitis (ASC) utilizing stacking ensemble learning framework and SHapley Additive exPlanations (SHAP) algorithm.

**Methods:**

This retrospective study was conducted on 492 patients with pathologically confirmed acute cholecystitis, collected from two tertiary hospitals between January 2020 and January 2023. The analysis was performed in December 2025. The patients divided into a training set (*n* = 413, Center 1) and an external test set (*n* = 79, Center 2). We developed a two-level stacking ensemble framework: level 1 employed attentive interpretable tabular network (TabNet) for CT radiomics and extreme gradient boosting (XGBoost) for clinical/imaging features as base learners; level 2 utilized logistic regression as a meta-learner to fuse predictions. The stacking model was compared with standalone TabNet and XGBoost models.

**Results:**

In five-fold cross-validation, the stacking model achieved a mean area under the curve (AUC) of 0.850, surpassing standalone TabNet (0.807) and XGBoost (0.799). This superiority was maintained in the external test set (AUC: 0.827 vs. 0.753 and 0.792). In the external test set, the stacking model also yielded the lowest Brier score (0.156) and the highest clinical net benefit in decision curve analysis. SHAP analysis identified neutrophil percentage, gallbladder wall necrosis, and pericholecystic exudation as the most influential clinical predictors, with radiomic features providing a higher overall weight in the final ensemble.

**Conclusions:**

The interpretable stacking model effectively integrates clinical and radiomic data to accurately predict ASC preoperatively.

## Introduction

According to the 2018 Tokyo Guidelines (TG18), grade III (severe) acute cholecystitis (AC) is defined as AC accompanied by one or more organ dysfunctions [1]. However, this grading system focuses more on systemic inflammatory response and treatment decision-making, rather than the severity of local gallbladder tissue damage. In clinical practice, some patients may not exhibit overt organ dysfunction, yet pathological examination reveals gallbladder wall necrosis or purulent destruction, and these patients tend to have worse outcomes than those with ordinary AC [2,3]. Therefore, this study focused on the severe inflammatory destruction of the gallbladder itself. We adopted a pathological definition widely accepted in imaging and surgical research, and based on that definition acute gangrenous cholecystitis (AGC) and acute purulent cholecystitis (APC) were classified together as acute severe cholecystitis (ASC) [4–8].

Compared with ordinary acute cholecystitis, patients with ASC not only have worse prognosis and higher mortality, but also experience significantly longer operative times and a markedly increased likelihood of conversion from laparoscopic to open surgery [9–11]. This is mainly because the more severe the local inflammation, the higher the surgical difficulty and the greater the risk of biliary injury [11,12]. Therefore, accurate preoperative assessment of the severity of gallbladder inflammation is of great clinical significance for formulating appropriate surgical strategies and improving operative safety. As a rapidly progressive and potentially life-threatening condition, ASC usually requires early intervention once diagnosed to improve patient outcomes [3,13]. Accurate preoperative assessment of ASC not only facilitates risk stratification but also directly influences surgical planning. Specifically, if ASC is identified preoperatively, surgeons may opt for safer alternative procedures, such as subtotal cholecystectomy, to avoid intraoperative decision-making under unfavorable conditions, thereby reducing the risk of complications such as bile duct injury [14,15]. Therefore, accurate preoperative identification of ASC can help optimize surgical planning, guide appropriate timing of intervention, and ultimately improve patient outcomes.

Currently, some studies have focused on the preoperative prediction of ASC [6,8,16,17]. However, these studies are mostly limited to traditional statistical models, such as logistic regression, and the predictive factors they construct largely rely on manually interpreted imaging features. They fail to fully exploit the deep quantitative information contained in medical images, which goes beyond what the human eye can discern.

In recent years, artificial intelligence (AI)-based predictive models have offered a new opportunity to address this challenge. Currently, most AI studies train models directly on raw images, making their decision-making process a ‘black box’ that is difficult for clinicians to understand and trust [18]. To overcome this limitation, our study employed radiomics analysis, a strategy widely recognized in the field, which transforms images into quantitative features with clear physical meaning and inherently provides a basis for model interpretability [19–21]. Building on this, we introduced TabNet, a deep learning model specifically designed for tabular data [22]. Unlike traditional ‘black-box’ deep learning models, TabNet uses its inherent attention mechanism to clearly indicate which key radiomic features contribute most to the prediction of ASC. This makes the model’s decision-making process transparent and interpretable, perfectly bridging the gap between advanced algorithms and clinical trustworthiness. For missing values commonly encountered in clinical data, methods such as multiple imputation are widely used to handle them. However, multiple imputation is sensitive to model specification, and improper handling may introduce bias, thereby affecting the stability and reliability of predictive results [23]. To address this, we chose the XGBoost model to handle clinical data. This model employs a sparsity-aware split finding algorithm, which can natively handle missing values by automatically learning the optimal direction for missing data at each node in the tree, without the need for prior imputation [24]. Finally, the predictive strengths of both models were combined using a stacking ensemble approach to develop a novel preoperative predictive model for ASC.

We hypothesize that this targeted fusion framework can capture the complementary value in multimodal information more effectively than simply combining all data and feeding it into a single model. By testing this hypothesis, the present study aims to provide clinicians with a more accurate and reliable tool for the preoperative identification of ASC among AC patients.

## Materials and methods

### Study population

**Figure 1.**
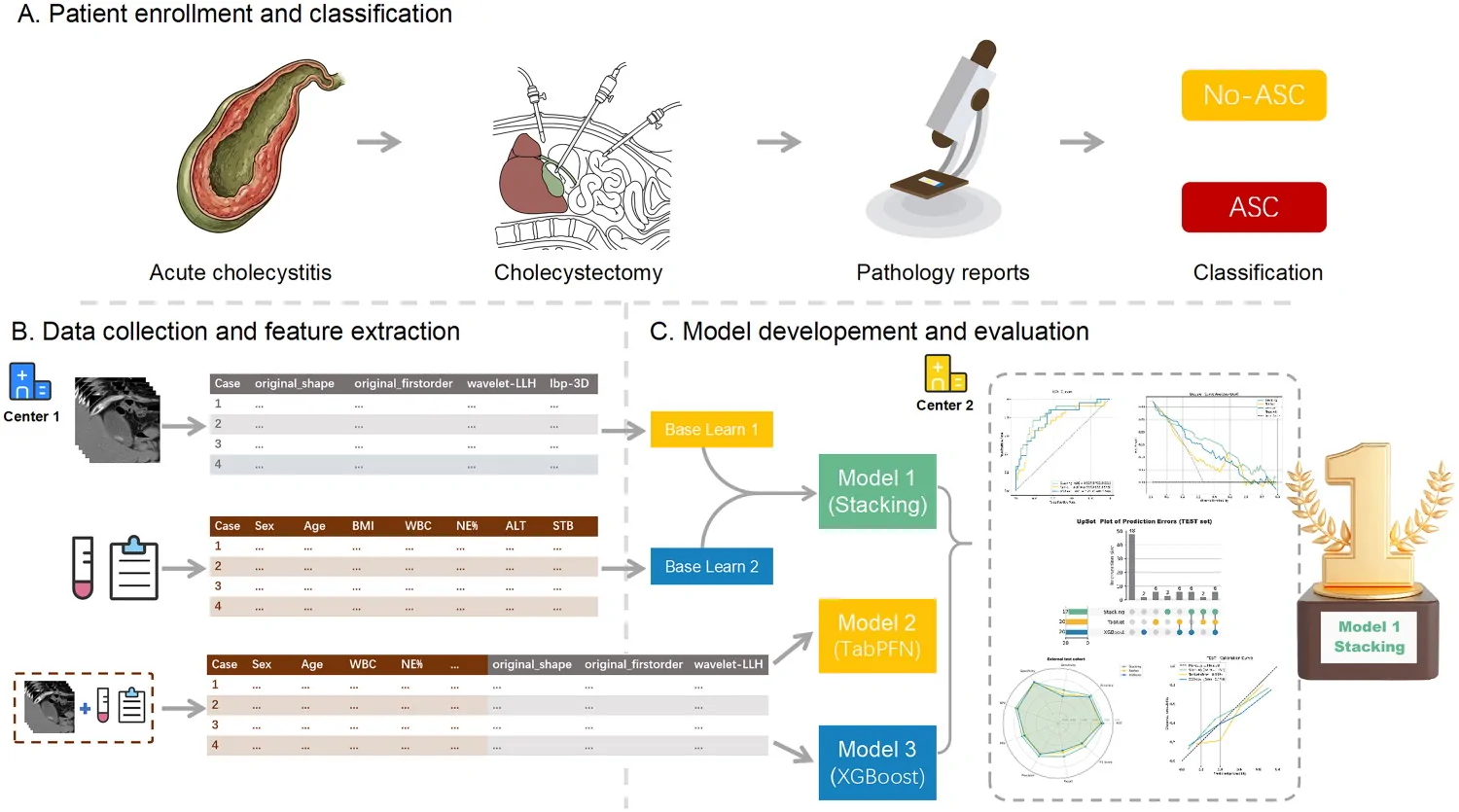
Diagram shows workflow (A–C). (A) Patient enrollment and classification. (B) Data collection and feature extraction. (C) Model developement and evaluation. ASC, acute severe cholecystitis.

This retrospective, two-center study was approved by the institutional review boards of the two participating hospitals and strictly adhered to the tenets of the Declaration of Helsinki (Center 1 Approval No. 2025K084; Center 2 Approval No. LLSC-2025-LW-01). Given the retrospective design, a waiver of participant consent was granted by the Institutional Review Board of the People’s Hospital of Liaoning Province, and the Institutional Review Board of Panjin Liaohe Oilfield Gem Flower Hospital. Inclusion criteria: (1) Patients who underwent cholecystectomy between January 2020 and January 2023; (2) Pathologically confirmed acute cholecystitis; (3) Complete laboratory data and contrast-enhanced CT scans performed within 48 h before surgery, with data available for analysis. Exclusion criteria: (1) Concomitant acute pancreatitis, cholangitis, or other abdominal emergencies requiring urgent intervention; (2) CT images with poor quality that were unsuitable for analysis. The study flowchart is presented in Figure 1.

### Definition and classification of ASC

ASC was defined based on postoperative pathological findings, including APC and AGC. According to the pathological results, patients were classified into the ASC group and the non-ASC (N-ASC) group, with the N-ASC group comprising those who did not meet the above criteria.

### Baseline clinical and radiologic characteristics

Baseline clinical characteristics included sex, age, body mass index (BMI), and a history of diabetes or hypertension. Laboratory indicators, obtained within 48 h preoperatively, included white blood cell count (WBC), neutrophil percentage (NE), alanine aminotransferase (ALT), and serum total bilirubin (STB). Imaging features were assessed based on abdominal CT scans performed as part of routine clinical evaluation after admission, including gallbladder (GB) stones, Stratification of bile in the lumen, gas within the GB lumen, necrosis of the GB wall, and pericholecystic exudation or fluid. All imaging features were independently evaluated by two experienced abdominal radiologists. Any discrepancies were resolved through consultation with a third senior radiologist, and the entire assessment process was conducted blinded to patients’ clinical information and pathological results. Furthermore, supplementary clinical context, including physical examination features and available ultrasound findings, is provided in Supplementary Tables S1 and S2.

**Table 1.** Baseline clinical and radiologic characteristics of patients.

Variable	Training cohort	External test cohort	*p* value
Age, (years)	66 (54, 75)	65 (55, 74)	0.747
BMI (kg/m²)	24.22 (22.04, 27.4)	24.46 (22.04, 26.35)	0.844
Sex			0.108
Male	208 (50.4%)	32 (40.5%)	
Female	205 (49.6%)	47 (59.5%)	
Diabetes	92 (22.3%)	12 (15.2%)	0.158
Hypertension	97 (23.5%)	14 (17.7%)	0.261
WBC (×10^9^/L)	9.64 (6.95, 13.83)	8.31 (5.54, 13.15)	0.059
NE (%)	80.90 (68.15, 89.10)	76.30 (62.40, 87.90)	0.053
ALT (U/L)	28.00 (17.00, 53.85)	23.00 (13.20, 42.30)	0.050
STB (μmol/L)	18.30 (12.45, 30.35)	15.30 (12.00, 25.60)	0.139
GB stones	255 (61.7%)	49 (62%)	0.108
Stratification of bile in the lumen	33 (8.0%)	4 (5.1%)	0.962
Gas within the GB lumen	20 (4.8%)	3 (3.8%)	0.366
Necrosis of the GB wall	95 (23.0%)	15 (19.0%)	>0.999^a^
Pericholecystic exudation or fluid	179 (43.3%)	34 (43.0%)	0.433
ASC	148 (35.8%)	26 (32.9%)	0.618

Abbreviations: BMI, body mass index, WBC, white blood cells, NE, neutrophil granulocytes, ALT, alanine aminotransferase, STB, serum total bilirubin, GB, gallbladder. Data are medians, with IQRs in parentheses.

^a^Fisher’s exact test.

### Radiomics feature extraction

One abdominal radiologist (Reader 1) manually delineated the entire gallbladder as the volume of interest (VOI) on non-contrast CT images using 3D-Slicer (v5.2.2). Detailed scanner information and acquisition parameters are provided in Appendix S1. To enhance feature robustness, images were resampled to 1 × 1 × 1 mm³ using nearest-neighbor interpolation, and intensities were discretized into 25 fixed bins. Radiomic features (first-order statistics, shape, and texture) were extracted using PyRadiomics, derived from original and filtered images (Wavelet, Logarithm, Square, LBP-3D, Gradient, Exponential, and SquareRoot). To assess inter-observer reproducibility, a second radiologist (Reader 2) independently segmented 30 randomly selected cases. Radiomic features were then extracted based on the VOIs from both readers, and the intraclass correlation coefficient (ICC) was calculated for each feature to quantify inter-observer reliability. Only features with ICC ≥0.75 were retained for subsequent feature selection and model development, while features below this threshold were excluded due to insufficient reliability.

All numerical features were standardized using *Z*-score normalization based on the training set. Categorical clinical features were encoded using one-hot encoding.

### Model construction

This study developed a stacking ensemble learning model composed of two base learners (TabNet and XGBoost) and a logistic regression meta-learner to integrate radiomic and clinical features for ASC prediction. The base models generated probability outputs, which were subsequently fused at the decision level to produce the final prediction. More detailed methodological and training information has been provided in the Appendix S2.

### Model comparison and evaluation

To evaluate the stacking ensemble model, two comparators were established. (a) TabNet model: The preprocessed clinical and radiomic features were combined and directly input into TabNet for end-to-end training. (b) XGBoost model: The combined feature set was first prefiltered. Significant features were selected using univariate analysis (*p* < 0.05), followed by correlation analysis (*r* < 0.9) to remove redundant features and ensure feature independence.

The model performance was comprehensively evaluated on the training set (using five-fold cross-validation) and the independent test set using multiple metrics. The area under the receiver operating characteristic curve (AUC) was used to assess overall discriminative ability. Classification performance was evaluated using accuracy, precision, recall (sensitivity), and F1 score. Finally, decision curve analysis (DCA) was performed to evaluate the clinical net benefit of each model across different decision thresholds. Differences in AUC between models were statistically compared using DeLong’s test. To enhance model interpretability, SHapley Additive exPlanations (SHAP) analysis was conducted to quantify feature contributions.

### Statistical analysis

Statistical analyses were performed using SPSS (v24.0) and Python (v3.10.9). Non-normally distributed continuous variables were expressed as median (interquartile range) and compared using the Mann–Whitney *U* test. Categorical variables were presented as counts (percentages) and compared using the chi-square test or Fisher’s exact test. All hypothesis tests were two-sided, and *p* < 0.05 was considered statistically significant. Model performance evaluation and visualization were conducted in the Python environment, primarily using libraries such as scikit-learn and Matplotlib.

## Results

A total of 492 patients with acute cholecystitis (AC) were included in this study, comprising a training cohort from Center A (*n* = 413) and an external test cohort from Center B (*n* = 79). The training cohort included 148 patients with ASC and 265 patients with N-ASC, while the external test cohort included 26 and 53 patients, respectively. The distribution of clinical variables was generally comparable between the two groups, and none of the differences reached statistical significance (all *p* ≥ 0.05), as summarized in Table 1.

**Figure 2.**
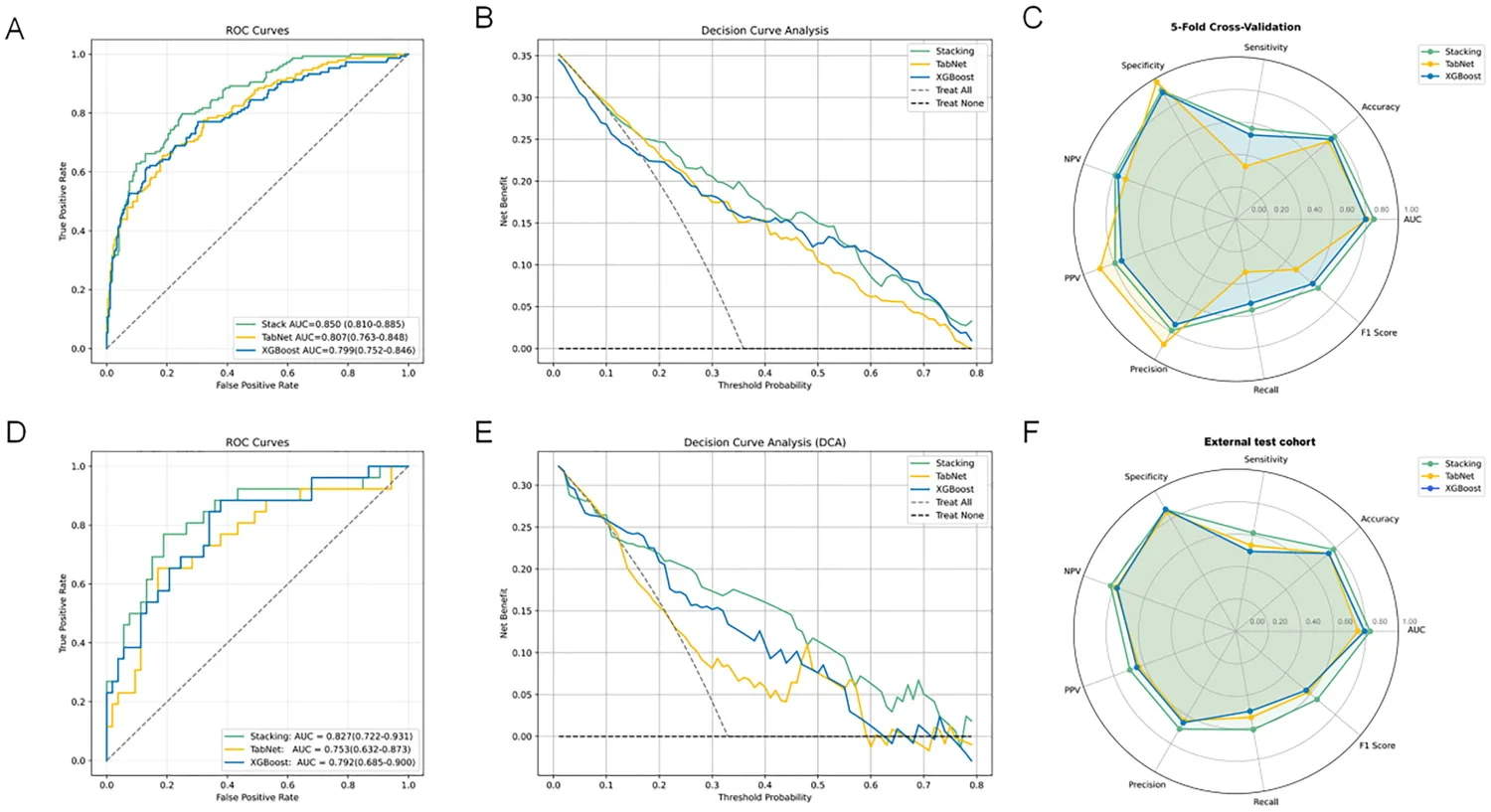
Performance of predictive models. (A,D) ROC curves showing discriminative ability of the models. (A) Five-fold cross-validation, (D) external test cohort. (B,E) Decision curve analysis (DCA) demonstrating net clinical benefit. (B) Five-fold cross-validation, (E) external test cohort. (C,F) Radar plots comparing performance metrics [accuracy, sensitivity, specificity, negative predictive value (NPV), positive predictive value (PPV), F1 score, AUC, etc.]. (C) Five-fold cross-validation, (F) external test cohort.

In the five-fold cross-validation of the training cohort, the stacking model achieved an AUC of 0.850 (0.810–0.885), which was higher than that of the TabNet model (AUC = 0.807 [0.763–0.848]) and the XGBoost model (AUC = 0.799 [0.752–0.846]) (Figure 2(A)). DCA in the training cohort showed that the stacking model provided the highest net benefit within a risk threshold range of 0.2–0.6 (Figure 2(B)). The radar chart indicated that the stacking model outperformed the baseline models in accuracy, AUC, F1 score, recall, sensitivity, and negative predictive value (NPV), while the TabNet model exhibited slightly higher positive predictive value (PPV), precision, and specificity compared to the other models (Figure 2(C)).

In the independent test cohort, the stacking model achieved an AUC of 0.827 (0.722–0.931), which was higher than that of the TabNet model (AUC = 0.753 [0.632–0.873]) and the XGBoost model (AUC = 0.792 [0.685–0.900]) (Figure 2(D)). Decision curve analysis (DCA) in the test cohort showed that the stacking model provided the highest net benefit within a risk threshold range of 0.2–0.7 (Figure 2(E)). The radar chart demonstrated that the stacking model outperformed the baseline models across accuracy, AUC, F1 score, recall, sensitivity, NPV, and PPV (Figure 2(F)).

**Figure 3.**
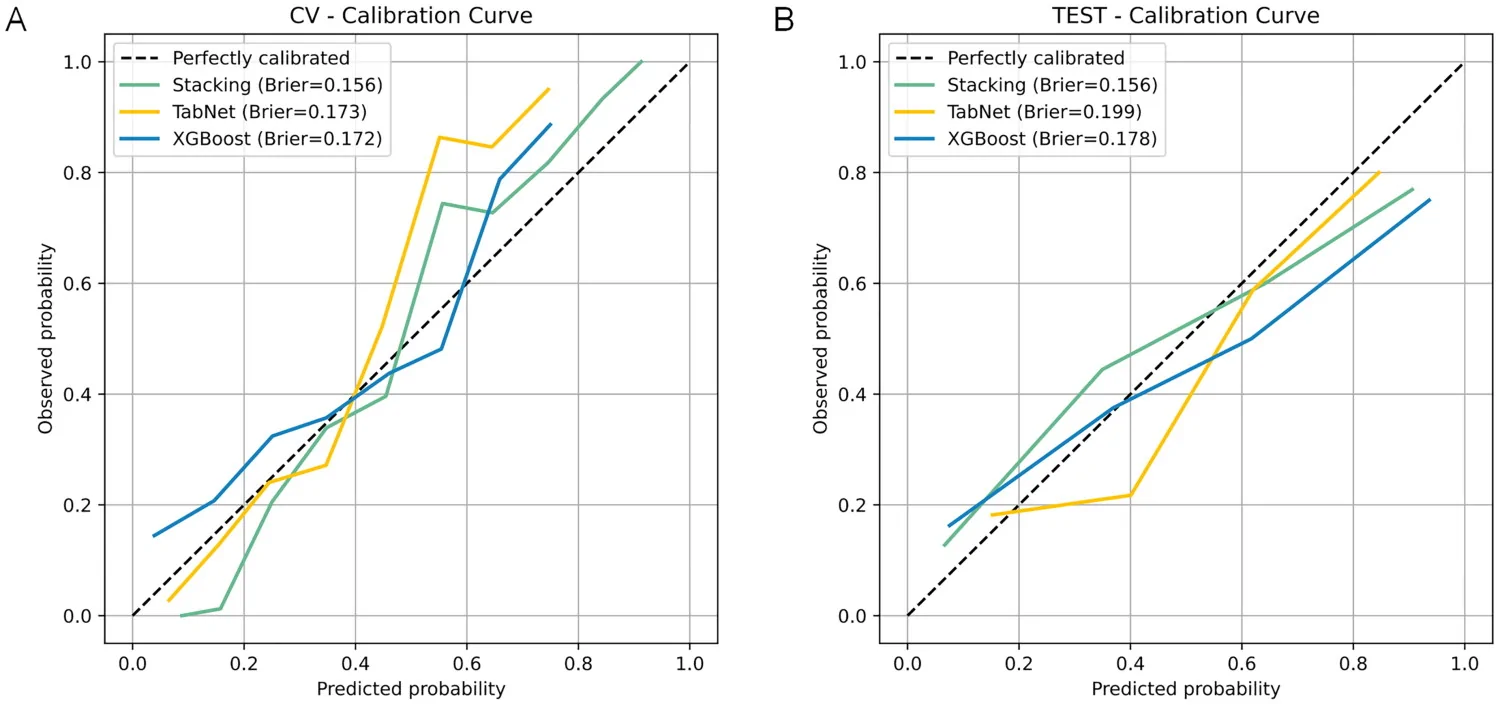
Calibration curves of predictive models. (A) Calibration performance in the 5-fold cross-validation (CV) set. (B) Calibration performance in the external test cohort.

Figure 3 presents the calibration curves for both the 5-fold cross-validation and the external validation datasets. During cross-validation, all three models exhibited good calibration performance, with Brier scores of 0.156 for the stacking model, 0.173 for TabNet, and 0.172 for XGBoost. In the external validation set, the stacking model continued to achieve the lowest Brier score (0.156), followed by XGBoost (0.178) and TabNet (0.199), indicating that stacking provides the most reliable probability estimates among the three models.

**Figure 4.**
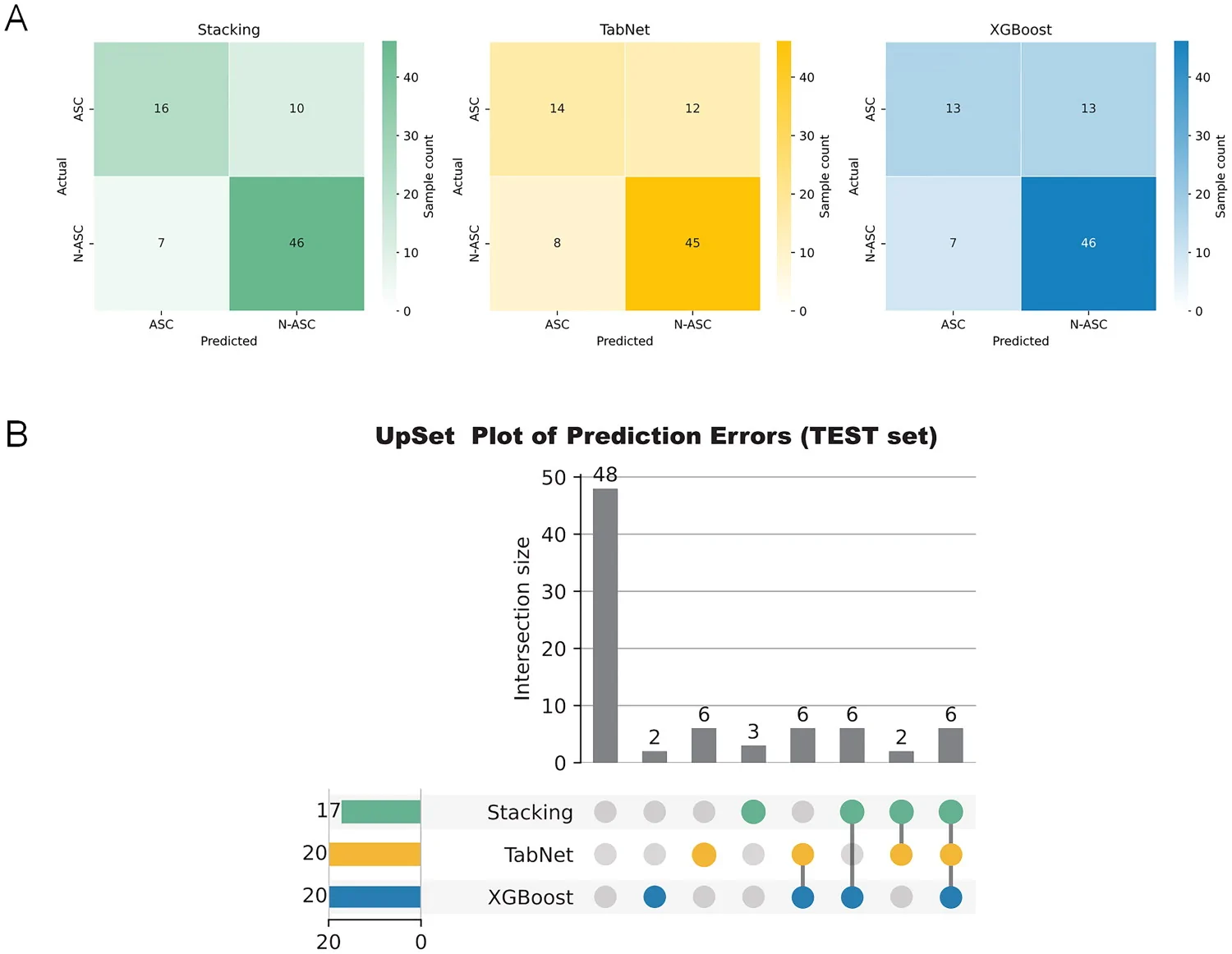
Performance evaluation of predictive models in the external test cohort. (A) Confusion matrices curves of predictive models. (B) UpSet plot of prediction errors across the predictive models. The horizontal bar chart on the left indicates the total number of misclassified samples for each model. The top vertical bar chart illustrates the intersection size of error sets, with the dot matrix below defining the specific combinations of models.

In the external test set, the confusion matrices (Figure 4(A)) indicated that the stacking model produced 10 false negatives (FN) and 7 false positives (FP). TabNet yielded 12 FN and 8 FP, while XGBoost had 13 FN and 7 FP. An Upset plot further illustrated the distribution of predictions across the three independent models (Figure 4(B)). Among the 79 test samples, 48 were correctly predicted by all three models, and 6 were mispredicted by all three. Three samples were mispredicted only by stacking, whereas 6 samples were correctly predicted exclusively by stacking. Similarly, TabNet mispredicted 6 samples alone and correctly predicted 5 samples uniquely. XGBoost mispredicted 2 samples alone and correctly predicted 2 samples uniquely.

**Figure 5.**
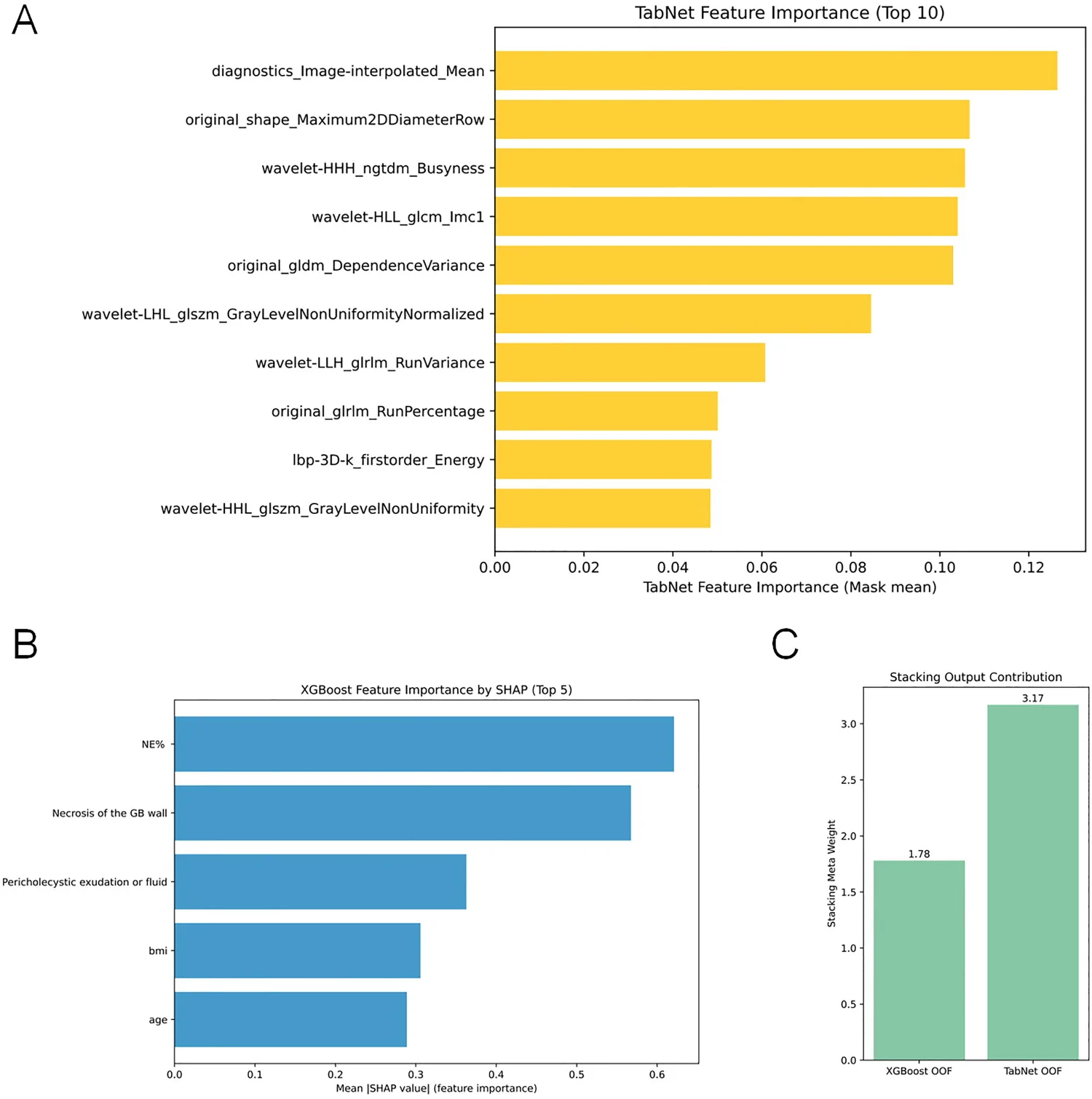
Interpretability analysis of the stacking ensemble model. (A) Feature contribution ranking of base learner 1 (top 10 features). (B) Feature contribution ranking of base learner 2 (top 5 features). (C) Weight distribution of base learner outputs in the stacking meta-learner.

We analyzed the feature contributions of its two base learners. In base learner 1, SHAP analysis revealed that among the top 10 most important features, ‘Wavelet-LHL_GLCM_Correlation’ and ‘Wavelet-LLL_Firstorder_Median’ contributed the most (Figure 5(A)). In base learner 2, the top five clinical variables were NE, necrosis of the gallbladder wall, pericholecystic exudation or fluid, BMI, and age (Figure 5(B)). In the meta-learner, logistic regression assigned weights of 3.17 and 1.78 to TabNet and XGBoost, respectively (Figure 5(C)).

## Discussion

The stacking model that integrated CT radiomics and clinical features outperformed each single algorithm, suggesting that multimodal information provides complementary value in the preoperative risk identification of AC. This finding indicates that, in complex inflammatory diseases, a single feature space may be insufficient to fully capture the underlying pathological heterogeneity, whereas ensemble strategies can more effectively integrate predictive signals from different domains. Our results are also consistent with recent studies showing the robust advantages of multimodal ensemble approaches in medical prediction modeling [25,26]. In addition, SHAP analysis of the base learners within the stacking framework revealed ten radiomics features with the highest contributions. Five clinical variables, namely NE, necrosis of the gallbladder wall, pericholecystic exudation or fluid, BMI, and age, were also identified as the most influential clinical predictors.

In the fusion model constructed in this study, TabNet contributed a larger proportion of the overall weight, suggesting that it assigned greater predictive importance to radiomics features for identifying ASC. Among the top ten features with the highest importance scores, most were related to imaging heterogeneity, which aligns well with the known radiologic and pathologic characteristics of ASC. First-order features such as mean and energy primarily reflected changes in the overall attenuation of the gallbladder lumen. Texture features including GrayLevelNonUniformityNormalized, GrayLevelNonUniformity, RunVariance, Imc1, and RunPercentage tended to capture irregularities in gray-level distribution, indicating potential structural disruption or mixed components within the gallbladder contents or wall. Previous imaging studies have shown that during the suppurative or gangrenous stage, the gallbladder lumen often contains purulent debris, desquamated mucosa, proteinaceous clots, and necrotic tissue, resulting in turbid, heterogeneous, or even layered and flocculent intraluminal appearances [27–29]. The mixture of these complex components causes the gray-level distribution to deviate from the uniform pattern of normal bile, thereby increasing heterogeneity at the texture level. The texture features identified in our study are largely consistent with this pathophysiologic understanding. An elevated busyness value indicates more frequent gray-level fluctuations among neighboring pixels, whereas an increase in DependenceVariance reflects a more dispersed pattern of gray-level dependence within local neighborhoods. These alterations are consistent with the coexistence of multiple pathological processes in severe cholecystitis, including mucosal necrosis, tissue fragmentation, stromal edema, and microhemorrhage [30,31]. Notably, previous studies have reported that gallbladder diameters exceeding 4.0 cm are often associated with more severe acute cholecystitis, supporting our observation [30].

In this study, NE was identified as the most important clinical predictor for ASC. This finding is consistent with previous studies, which have reported that the risk of severe cholecystitis increases with higher NE levels [32]. An elevated neutrophil count indicates an active acute inflammatory response and is often associated with the degree of gallbladder wall necrosis [33]. In addition, necrosis of the GB wall and pericholecystic exudation or fluid, as selected by the model, were identified as important imaging predictors of ASC. Previous studies have also recognized these two changes as potential indicators for predicting ASC [34,35]. Notably, although baseline BMI levels were similar between patients with ASC and N-ASC, the model still identified it as a valuable predictive factor. We speculate that BMI may influence severe outcomes not through an independent effect. it may act by modulating or amplifying the association between other key features, such as neutrophil percentage, and severe outcomes. Age was identified as an important predictor in our model. This finding is consistent with previous studies, which have recognized age as a risk factor for ASC [36,37].

According to TG18, WBC is an important indicator for assessing the severity of cholecystitis [1]. Multiple studies have also suggested that an elevated WBC count is a risk factor for ASC [30,32,38]. However, in our study, WBC ranked relatively low in terms of feature importance.

This may be because WBC is highly correlated with NE. When handling closely related features, the model tends to retain the one most strongly associated with the disease. As a result, the relative contribution of WBC is reduced. Although acute acalculous cholecystitis is relatively uncommon, it progresses rapidly and can be severe. Literature reports that approximately 40%–50% of patients may develop serious complications such as gangrene, empyema, or perforation [39]. Therefore, theoretically, the presence of gallstones should be considered an important predictive factor. After discussion, we believe this may be because the presence of gallstones in our study was assessed using CT, which has limited sensitivity for cholesterol stones. As a result, the ‘gallstone’ feature in this study only represents the types of stones detectable by CT. This suggests that cholesterol stones may be an important predictor of severe cholecystitis. Future studies should incorporate imaging modalities more sensitive to cholesterol stones, such as ultrasound, to analyze the relationship between different types of gallstones and the risk of ASC.

Despite dual-center validation enhancing reliability, this study has several limitations. First, all patients included in this study underwent cholecystectomy. Compared with those who received conservative treatment, these patients may have had more severe illness or more difficult diagnoses, thus introducing a selection bias toward severe cases. Meanwhile, the validation sample size from Center 2 is relatively limited, and prospective, large-scale multicenter studies are still needed to confirm clinical utility. Second, all radiologic features were extracted from non-contrast CT scans which might omit key information available on contrast-enhanced scans, such as wall perfusion. Third, ultrasound serves as the first-line imaging modality in routine clinical management of AC; however, this study failed to incorporate its signs, as well as other potential predictors such as C-reactive protein, thereby limiting the model’s utility in real-world clinical screening. The absence of these variables stems from the retrospective study design, and future prospective studies are expected to systematically collect such indicators to further enhance the completeness of the model. Finally, this study did not incorporate intraoperative complexity or pathological grading into the proposed severity assessment. Future studies should integrate multi-source data to improve the accuracy of severity evaluation.

## Conclusions

We developed a stacking ensemble model integrating CT radiomics and clinical features for preoperative prediction of ASC. SHAP analysis identified SHAP analysis identified NE, necrosis of the GB wall, pericholecystic exudation or fluid, BMI and age as key predictors. By combining readily obtainable clinical indicators with radiomics features, the model may assist emergency and surgical physicians in more accurately identifying patients at risk of ASC. These patients may require timely surgical intervention and comprehensive preoperative preparation, thereby enabling optimized perioperative management.

## Supplementary Material

TableS.docx

AppendixS.docx

## Data Availability

The datasets used and/or analysed during the current study are available from the corresponding author on reasonable request.
