# Supplementary material for: Preoperative prediction of acute severe cholecystitis using an attentive interpretable tabular network (TabNet)-based radiomics model and a stacking ensemble: a two-center study
Source: Ann Med. 2026 Sep 15;58(1):2732512. doi: 10.1080/07853890.2026.2732512 (PMC13580380; doi:10.1080/07853890.2026.2732512)
Supplement: Supplementary file 1 [file IANN_A_2732512_SM2024.docx]

**Supplementary Material**

**Appendix S1: scan parameters of CT**

Center 1: The Siemens device utilized a tube current of 312 mA, tube voltage of 120 kVp, matrix size of 512×512, and a field of view (FOV) of 50 cm. The Toshiba device utilized a tube current of 300 mA, tube voltage of 120 kVp, matrix size of 512×512, and a FOV of 50 cm.

Center 2: The Siemens device utilized a tube current of 260 mA, tube voltage of 120 kVp, matrix size of 512×512, and a FOV of 50 cm.

**Appendix S2: Model construction**

This study employed a Stacking ensemble learning framework to predict ASC by integrating radiomic and clinical features. The framework consisted of two levels. At the first level, two base learners were trained in parallel. Base learner 1 used the TabNet model, taking ICC-filtered and standardized radiomic features as input. Base learner 2 used the XGBoost model, taking standardized clinical features as input. Both base learners were trained under the same five-fold cross-validation splits, generating out-of-fold (OOF) predicted probabilities. At the second level, the OOF predictions from the two base learners were concatenated to form a new feature matrix, which was then input into a logistic regression model as the meta-learner. This decision-level feature fusion yielded the final Stacking ensemble predictive model.
