# Supplementary material for: Preoperative prediction of acute severe cholecystitis using an attentive interpretable tabular network (TabNet)-based radiomics model and a stacking ensemble: a two-center study
Source: Ann Med. 2026 Sep 15;58(1):2732512. doi: 10.1080/07853890.2026.2732512 (PMC13580380; doi:10.1080/07853890.2026.2732512)
Supplement: Supplementary file 2 [file IANN_A_2732512_SM2025.docx]

**Table S1** Preoperative ultrasound features of 134 patients with acute cholecystitis

| Ultrasound features | ASC (n = 41) | Non-ASC (n = 93) |
| --- | --- | --- |
| Pericholecystic fluid | 24 (58.5%) | 32 (34.4%) |
| Gallbladder distention | 29 (70.7%) | 61 (65.6%) |
| Edematous gallbladder wall | 32 (78.0%) | 59 (63.4%) |
| Gallstones or sludge | 34 (82.9%) | 73 (78.5%) |

ASC acute severe cholecystitis.

**Table S2** Physical exam features of patients with acute cholecystitis

| Physical exam features | ASC (n = 174) | Non-ASC (n = 318) | *P* value |
| --- | --- | --- | --- |
| Abdominal pain | 168 (96.5%) | 312 (98.1%) | 0.360* |
| RUQ pain | 134 (77.0%) | 254 (79.9%) | 0.457 |
| Murphy's sign | 112 (64.4%) | 219 (68.9%) | 0.309 |

ASC acute severe cholecystitis. * Fisher’s exact test. RUQ, Right upper quadrant.
